# Feeling Supported as a Remote Worker: The Role of Support from Leaders and Colleagues and Job Satisfaction in Promoting Employees’ Work–Life Balance

**DOI:** 10.3390/ijerph21060770

**Published:** 2024-06-14

**Authors:** Ilaria Buonomo, Clara De Vincenzi, Martina Pansini, Francesco D’Anna, Paula Benevene

**Affiliations:** Department of Human Sciences, LUMSA University, 00193 Rome, Italy; i.buonomo1@lumsa.it (I.B.); c.devincenzi2@lumsa.it (C.D.V.); f.danna3.dottorati@lumsa.it (F.D.);

**Keywords:** work–life balance, job satisfaction, COR theory, social support, remote working

## Abstract

Due to the increasing use of remote work, understanding the dynamics of employee support and its implications for job satisfaction and work–life balance is crucial. Utilizing the Conservation of Resources (COR) theory as a theoretical framework, this research investigated how feeling supported by leaders and colleagues at work fosters work–life balance and job satisfaction among remote employees. The study involved 635 remote workers (females = 61%, mean age, 46.7, SD = 11) from various service-based industries and public administration in Italy. Results from the structural equation model showed a total mediating effect of job satisfaction in the link between colleague support and work–life balance (χ^2^_(22)_ = 68.923, *p* = 0.00, CFI = 0.973, TLI = 0.955, RMSEA = 0.059 (90% CI = 0.044–0.075, *p* = 0.158), SRMR = 0.030), emphasizing the role of interpersonal relationships within the workplace in enhancing remote workers’ job satisfaction and, consequently, their work–life balance. Contrary to expectations, the study found no significant direct or indirect link between leader support and work–life balance. This research highlights the significance of fostering strong social connections and ensuring employee satisfaction to promote well-being and work–life balance in remote work arrangements.

## 1. Introduction

Depending on how they are managed, new ways of working and new technologies at work can have a wide range of effects. The introduction of remote working has changed the paradigm of the conventional office to more dynamic and diverse environments, offering new perspectives on employee engagement and organizational structure [1]. Several studies have shown that remote working conditions can lead to lower stress and burnout, reduced home–work conflict, and higher engagement and satisfaction [2]. However, these benefits are particularly true when employees feel that their remote work is supported. With the rise of remote work, many employees are now facing new challenges related to social isolation, lack of connection with colleagues [3], and difficulty managing work–life balance [4].

In recent years, there has been growing interest in understanding the impact of social support at work on the well-being of remote workers. Social support at work can be defined as the resources and assistance provided by colleagues and supervisors to buffer against the harmful effects of work-related stressors. Bentley and colleagues [5] showed that the perceived organizational support in remote working conditions prevents strain conditions more significantly than the remote arrangement itself. However, other studies (e.g., [6,7]) have shown that mental health risks, such as stress, fatigue, and burnout, can occur during teleworking, especially when there is a lack of perceived support from colleagues [8]. Not feeling supported can lead to a heightened risk of stress and anxiety [9], higher feelings of isolation [10], and a lower sense of belonging to the organization [11].

The existing literature on remote work arrangements and their effects on employee well-being shows that it is not the work arrangement itself, but rather the conditions provided by the organizations to plan and support employees working remotely, that impact how employees understand and experience remote working [12]. For example, Adamovic [13] conducted a study with administrative and teaching staff at Iraqi universities, showing that employees who do not believe that telecommuting will lead to social isolation experience a reduction in job stress.

The impact of teleworking on employees’ relationships regarding private life is also important to consider. Generally speaking, working from home can blur the boundaries between work and family because it is more challenging to separate work time from family time [14]. Current studies on the impact of teleworking or remote working arrangements on work–life balance provide contradictory findings. Some studies highlight positive outcomes, such as reduced conflict between work and home responsibilities on telecommuting days [15], often attributed to decreased time pressure associated with remote work arrangements (e.g., [16]). However, other studies indicate a negative relationship between telecommuting conditions and work–life balance, reporting increased conflict between the two domains (e.g., [17]), as supported by longitudinal studies (e.g., [18]).

Despite extensive research on remote work’s benefits, significant gaps persist in understanding how specific forms of social support from colleagues and leaders impact remote employees’ well-being. Previous studies have largely focused on the general effects of perceived organizational support, neglecting the nuanced roles of support from colleagues versus leaders. Additionally, the literature lacks depth on how this support translates into tangible outcomes, such as job satisfaction and work–life balance. This study addresses these gaps by examining the distinct influences of colleague and leader support, using job satisfaction as a key mediator. This approach extends the application of the Conservation of Resources (COR) theory to remote work dynamics and elucidates the pathways through which different forms of social support affect employee outcomes, thereby filling a critical void in the existing literature.

The following sections will describe our research objectives, hypothesized relationships between variables, and the theoretical framework guiding our study. Thus, this study proposes to explore the differential effects of support from colleagues versus leaders on remote workers’ job satisfaction, aiming to detail the unique contributions of these sources of support. Additionally, we focus on job satisfaction as a mediator to understand how it bridges the effects of social support on achieving a better work–life balance. This aspect is particularly crucial for managing remote work effectively and fostering an environment where employees can thrive both professionally and personally.

Thus, this research aims to deepen our understanding of how different forms of social support—specifically from colleagues and leaders—affect remote employees’ job satisfaction and work–life balance. We aim to understand how variations in support from these two distinct sources can uniquely contribute to enhancing the work conditions for remote employees.

### 1.1. Literature Review, Research Hypothesis, and Conceptual Model

The existing literature provides evidence of the helpful impact of perceived support at work on remote employee well-being and the extension of the effects of remote work arrangements on employees’ perceived quality of relationships in different life domains. Building on these considerations, this study aims to investigate whether and how feeling supported by colleagues and leaders at work promotes work–life balance among remote employees.

Specifically, this work seeks to understand how social support in remote work arrangements can be a valuable resource for employees, helping them manage the demands of work and their personal lives more effectively. Work–life balance refers to the ability of employees to balance their work responsibilities with their personal lives, including family, social, and leisure activities [19]. It is characterized by a sense of control over one’s time and the ability to manage competing demands between work and personal life.

By exploring the role of job satisfaction as a mediator, this study aims to shed light on the mechanisms through which perceived support at work can impact employee well-being and work–life balance. Job satisfaction, intended in this paper to be a proxy for work-related well-being, refers to an employee’s overall feelings and attitudes towards their job, including feelings of fulfillment, contentment, and motivation (see [20] for a thorough review of the construct).

In summary, this study aims to contribute to the growing body of literature on remote work arrangements, social support, and employee well-being by examining the relationship between perceived support at work, job satisfaction, and work–life balance for remote workers. By doing so, it is likely that valuable insights could arise for organizations seeking to support their remote workforce and promote employee well-being in these new and evolving work arrangements.

In the Conservation of Resources (COR) theory, resources are broadly defined and can include anything that individuals value. This might include personal characteristics (e.g., resilience, skills), tangible assets (e.g., a home office), social resources (e.g., support from colleagues or leaders), and conditions (e.g., job security, work–life balance). According to COR theory, individuals engage in constant efforts to gain new resources, keep the resources they already have, and safeguard against the loss of these resources. This dynamic is crucial for understanding employee behavior, especially in remote settings. For example, remote workers might seek to develop new skills (obtain) to enhance job security, strive to maintain a good relationship with their team (retain), and avoid overwork to protect their health and well-being (protect). Thus, COR theory can serve as a valuable guide for our study that focuses on the understanding of how the sense of support received from supervisors and colleagues in the workplace influences the attainment of work–life balance and increases job satisfaction among employees working remotely.

In the context of remote work, employees may experience a loss of social support due to physical distance and reduced opportunities for face-to-face interaction. However, if remote employees feel supported by their colleagues and leaders, they are more likely to view their work arrangement as a resource gain rather than a loss, thus promoting their work–life balance and job satisfaction. Moreover, the COR theory suggests that there is a permeability between the different domains of life, meaning that when employees receive sufficient support at work and are satisfied with their jobs, they have more resources to devote to other areas of their lives, including their private lives.

Overall, the application of COR theory to remote work contexts makes it an insightful theoretical framework for capturing the relevance of preserving and acquiring resources in sustaining and improving employee well-being and productivity, in terms of job satisfaction and work–life balance.

### 1.2. Colleagues and Leaders’ Support and Work–Life Balance

The existing literature on remote work arrangements and their impact on work–life balance suggests that remote working can increase the flexibility of work operations and promote work–life balance [21]. However, this is only possible when organizations provide adequate support to their employees, encourage them to feel a sense of control, and implement work–life balance strategies [22,23]. Studies have also shown that the joint effect of spousal support at home and coworkers’ support at work greatly contributes to perceived work–life balance [4,24]. The COVID-19 pandemic has further highlighted the critical role of organizational support in promoting employees’ work–life balance [4].

A plausible explanation for the link between feeling supported by coworkers and supervisors and experiencing better work–life balance is that a supportive social environment at work can encourage employees to communicate their needs and preferences more effectively. This can lead to greater flexibility in work arrangements and improved work–life balance. Overall, the evidence suggests that social support at work is a crucial factor in promoting work–life balance for remote workers.

Building on these considerations, we hypothesized the following:

**H1a.**  *Colleague support is associated with work–life balance.*

**H1b.**  *Leader support is associated with work–life balance.*

### 1.3. Colleagues and Leaders’ Support and Job Satisfaction

Remote work can be isolating and lonely, making it crucial for remote workers to feel supported by their colleagues and supervisors. Perceived support from colleagues can help remote workers feel more connected and engaged in their work, leading to higher job satisfaction. Studies have shown that social support at work acts as a coping mechanism, promoting positive adaptation to remote job demands [25,26,27]. Several studies have showed that social support from management and contact with colleagues play a crucial role in mitigating feelings of isolation and loneliness [28]. Feeling supported in using personal strengths at work can help buffer insecurities caused by technology [29], while perceiving support from colleagues can reduce feelings of strain and fatigue from technology use [30].

Feeling supported can also shape the way job demands are perceived, leading to higher job satisfaction. For example, Califf and colleagues [31] found that feeling supported when addressing challenging aspects of remote working, such as workload and time urgency, can contribute to a higher sense of job satisfaction. The positive link between perceived support and well-being measures, including job satisfaction, is confirmed when addressing the role of leaders. Research shows that the higher the perceived leader support, the higher the job satisfaction in remote working conditions [5].

While different leadership styles can impact job satisfaction in remote work, studies suggest that task-oriented leaders can be particularly beneficial for teleworkers [32]. Technical and structural support are also crucial for remote workers [33]. Transformational leadership is another style that has shown a positive impact on remote workers’ job satisfaction [34]. This is consistent with studies on in-office workers, which showed a significant indirect relationship between authentic leadership and followers’ job satisfaction through their perception of their leaders’ work–life balance and their own lives [35]. Overall, the evidence suggests that feeling supported by colleagues and supervisors is crucial for promoting job satisfaction in remote workers.

**H2a.**  *Colleague support is associated with job satisfaction for remote working.*

**H2b.**  *Leader support is associated with job satisfaction for remote working.*

### 1.4. Job Satisfaction and Work–Life Balance

While some studies have focused on the link between work–life balance and job satisfaction in remote working conditions (e.g., [36,37]), it is also important to consider the potential influence of job satisfaction on work–life balance. There are several reasons why job satisfaction may impact work–life balance positively. First, satisfied employees are more likely to be engaged and committed to their work and personal responsibilities [38,39]. This can be due to the broadening and building effect of positive emotions, which suggests that people who acknowledge the pleasantness of events and conditions in their lives are more likely to behave and think effectively [40].

Job satisfaction, as a proxy for work-related well-being, can also promote a lower risk of stress and burnout, thus enabling remote workers to deal more effectively with balancing work and personal demands [41]. This is particularly important in remote working conditions, where the boundaries between work and personal life can be blurred. Feeling satisfied with one’s job can provide a buffer against the potential stress and burnout that can arise from working remotely, leading to a higher chance for remote workers to achieve a better work–life balance.

Overall, while the relationship between work–life balance and job satisfaction in remote working conditions is important to consider, it is also crucial to recognize the potential influence of job satisfaction on work–life balance. Satisfied employees are more likely to be engaged, committed, and equipped to deal with the demands of balancing work and personal life, leading to a better work–life balance.

**H3.**  *Job satisfaction for remote working is associated with work–life balance.*

### 1.5. The Mediating Effect of Job Satisfaction for Remote Working

Job satisfaction can play a crucial role in mediating the link between social support at work and work–life balance for remote workers. When employees receive support from their colleagues and leaders while working remotely, they may feel happier and more satisfied with their jobs, as well as more valued and respected [21,23,42]. This can lead to increased job satisfaction, which can increase the likelihood of remote workers feeling in control of their time and ability to balance multiple roles in their different life domains, thus helping them manage the demands of their work and personal life more effectively [38,39].

Building on these considerations, we hypothesized the following:

**H4.**  *Job satisfaction for remote working mediates the relationship between colleague support and work–life balance.*

**H5.**  *Job satisfaction for remote working mediates the relationship between leader support and work–life balance.*

**Figure 1:**
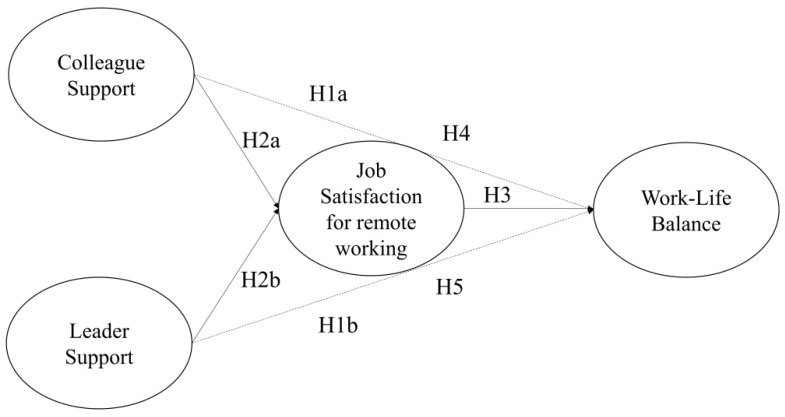
Theoretical model. Note. The dashed lines indicate the direct effects of colleague support and leader support on work–life balance. H4 and H5 refer to the mediation effect of job satisfaction for remote working in the effects of colleague support and leader support on work–life balance.

Finally, Figure 1 shows all the hypotheses in our model.

## 2. Methods

### 2.1. Participants and Procedures

This research included 635 Italian workers from several occupational sectors such as the research field (45%), service-based industry (finance, energy, legal services) (32%), and public administration (23%).

Most of the sample was composed of women (61%), with the rest (39%) composed of men. The sample’s age ranged between 21 and 70, the average age was 46.7 years (SD = 11), and the average years of experience in the current organization were 15.13 years (SD = 11.60). In this study, we adopted specific inclusion and exclusion criteria to select a representative sample of employees within organizations that facilitate flexible work arrangements.

Inclusion Criteria: We included employees who had the opportunity to work remotely and on-site, thus allowing an analysis of the impact of such flexible working modalities on employees’ well-being and effectiveness. Additionally, we selected participants without direct contact with clients, focusing on roles that exclude face-to-face interactions with customers, patients, or the public. Importantly, our sample also comprised employees on temporary or part-time contracts and full-time, to ensure a comprehensive analysis of organizational impacts across different employment statuses.

Exclusion Criteria: Workers occupying front-line roles or having direct interactions with the public, such as customer service representatives or front-line healthcare staff, were excluded to focus on internal organizational dynamics free from specific stressors associated with customer contact.

Informed consent was obtained from all subjects involved in the study before the administration of the questionnaire. The study was conducted in accordance with the Declaration of Helsinki and approved by the Ethics Committee of LUMSA University (protocol code 4/2022 on 28 October 2022).

Participants were informed about the responsibility, the purpose, and the procedure of the study, the content of the questionnaire that would be administered, as well as the anonymity and confidentiality of the data collected. Finally, exclusive access to the data was reserved for the research team, and information would not be disclosed to managerial staff. Such precautions were instituted to mitigate the risk of social desirability bias in participants.

### 2.2. Measures

A questionnaire was administered to assess various aspects of employees’ experiences with remote working. Using the Copenhagen Psychosocial Questionnaire (COPSOQ III) [43], each subscale was carefully translated and adapted from its original version, which was designed for traditional on-site work settings, to better capture the challenges and dynamics of remote work conditions.

Leader support (LS) was measured by the adapted COPSOQ III’s subscale of “Social support from the supervisor”. This adjusted subscale consists of three items aimed at evaluating the frequency and quality of support and assistance that remote employees receive from their immediate superiors. A key question from this scale is as follows: “In the setting of remote work, to what extent do you receive timely assistance and support from your superior when it’s needed?” Responses were scored on a Likert scale ranging from 0 (Never) to 5 (Always), with higher scores indicating a stronger support system provided by leaders to remote employees.

Colleague support (CS) was determined through the adaptation of the “Social support from colleagues” subscale from the COPSOQ III. This scale, with three items, assesses the level of help and camaraderie that remote workers experience from their peers. An illustrative question from this measure is as follows: “How frequently do your colleagues offer you help and support when you are working remotely and need it?” Responses are collected using a Likert scale from 0 (Never) to 5 (Always), where higher scores denote more robust support and interaction among colleagues in a remote working arrangement.

Job satisfaction (JS) was evaluated by revising the “Job satisfaction” subscale from the COPSOQ III to specifically address aspects of remote working. The adapted scale, containing two items, was designed to survey an employee’s satisfaction and well-being with his or her remote working arrangements (e.g., “How much are you satisfied with your remote job experience in general, taking everything into consideration?”). The responses were recorded on a Likert scale from 0 (Very unsatisfied) to 5 (Very satisfied), reflecting the degree of job satisfaction in remote settings.

Work–life balance (WLB) during remote working arrangements was measured by one item measuring the positive impact of remote working on private life organization (“The remote working conditions allowed for a better organization of my private life”). The Likert scale ranged from 0 (To a very small extent) to 5 (To a very large extent), where a higher score indicates a greater perceived balance between professional and personal life.

### 2.3. Plan of Analysis

This research was conducted as a cross-sectional study. This method allows for data analysis from a diverse population at a specific moment, which is particularly useful for identifying immediate effects within organizational settings.

The starting point was the data exploration following three procedures: uni- and multivariate outlier analysis using Mahalanobis’s distance thresholded at *p* < 0.001; an analysis of score distribution with skewness and kurtosis limits set between [−2; +2]; and an evaluation of missing values, which were excluded on a listwise basis. Following these steps, we procured the sample detailed previously.

In the preliminary analysis phase (with IBM-SPSS v. 24), Pearson’s correlations were measured between support from colleagues, support from the leader, job satisfaction, and work–life balance to analyze the associations between the constructs and demographics variables (age, gender, years of experience). Additionally, ANOVA (Analysis of Variance) tests were performed to examine potential differences across the segmented three ranges of age groups and years of experience within the organization. This approach investigated whether demographic differences significantly affect the variables of interest.

The next steps were dedicated to verifying the hypothesized measurement model.

To this end, we conducted a Confirmatory Factor Analysis (CFA) [44] using MPlus version 8 [45]. The Robust Maximum Likelihood Approach (MLR) was used to deal with non-normality in data [46]. To verify the validity of the model’s metrics, we calculated both the average variance extracted (AVE) and the composite reliability (CR) (refer to Table 1). An AVE higher than 0.50 and a CR lower than 0.70 indicates a satisfactory convergent validity [47]. Discriminant validity was tested through the Fornell–Larcker Criterion according to which the square root of the AVE of each construct should be higher than the construct’s highest correlation with any other construct in the model [48].

The structural equation modeling (SEM) [44] was then applied. Our model assumed that job satisfaction mediates, directly and indirectly, the relationship between support from colleagues and support from the leader and work–life balance. To assess the model’s fit, we used a comprehensive set of indices [49]. First, the Chi-square likelihood ratio statistic might be significant due to its sensitivity to sample size [44]. Next, the Tucker and Lewis Index (TLI), and the Comparative Fit Index (CFI) were used with values greater than 0.95 as significant [45]. Finally, the Root Mean Square Error of Approximation (RMSEA), with its confidence intervals, and the Standardized Root Mean Square Residual (SRMR) with lower than 0.08 as significant were also used [50,51].

## 3. Results

Table 1 reports the AVE and CR for each variable assessed in the study.

**Table 1:** Convergent validity and internal reliability.

Variables	AVE	CR	α	VIF
Support from colleagues	0.422	0.685	0.687	1.48
Support from leader	0.685	0.867	0.86	1.41
Job satisfaction	0.763	0.866	0.814	1.07

Note. AVE = Average Variance Extracted, CR = Composite Reliability, VIF = Variance Inflation Factor, α = Cronbach’s alpha.

Assuming that AVE values exceeding 0.50 and CR values surpassing 0.70 indicate robust constructs, the reported values of the variables demonstrate their reliability, except for support from colleagues, although still close to the cut-offs proposed. The variables also report a satisfactory internal variability (α > 70) [52]. Finally, all variables showed VIF values below 10 and tolerance levels above 0.1, indicating that multicollinearity is not a concern and thus validating the integrity of the model [53,54].

Regarding the discriminant validity (Table 2), the square root of the AVE of each construct shows higher values than the variable’s highest correlation with any other variable in the model.

Table 3 lists the means, standard deviations, and correlations for all the variables in the study that are essential for further analysis. Additionally, the ANOVA test reveals no significant differences according to participants’ age and years of experience.

**Figure 2:**
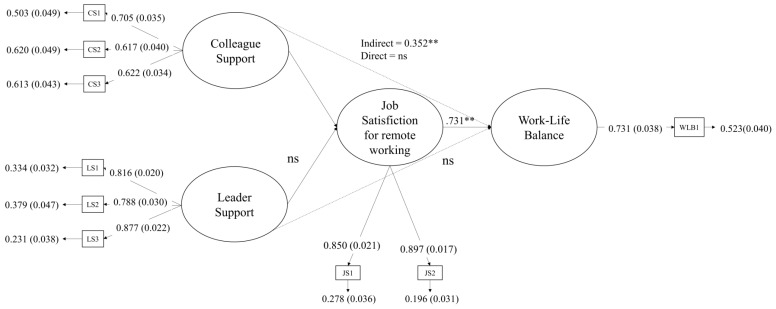
Final model. Results of the structural equation model. Standardized effects and significance values are reported. Note. ** = *p* < 0.01, ns—not significant. WLB—Work–life balance; SC—Support from colleagues; SL—Support from the leader; JS—Job satisfaction for remote working.

Results from the structural equation model (Figure 2) showed a total mediating effect of job satisfaction in the link between colleague support and work–life balance (χ^2^_(22)_ = 68.923, *p* = 0.00, CFI = 0.973, TLI = 0.955, RMSEA = 0.059 (90% CI = 0.044–0.075, *p* = 0.158), SRMR = 0.030).

All the mentioned hypotheses were confirmed, except H1b (leader support is associated with work–life balance), H2b (leader support is associated with job satisfaction for remote working), and H5 (job Satisfaction for remote working mediates the relationship between leaders support and work–life balance). When taking into account the mediating effect of job satisfaction for remote working, the direct association between colleague support and work–life balance was not significant (H1a; βdirect = −0.111, ns), thus showing a total mediating effect (H4; βindirect = 0.352, *p* < 0.01).

## 4. Discussion

The present study addressed the connections between perceived support from colleagues and leaders and its association with work–life balance among remote workers, with a particular focus on the role of job satisfaction as a potential mediator. The findings shed light on nuanced relationships, only partially confirming our hypotheses.

Specifically, the results confirmed that colleague support is positively associated with both work–life balance (H1a) and job satisfaction (H2a), supporting the significant role of peer interactions in enhancing remote working conditions. Similarly, job satisfaction was found to be a significant mediator in the relationship between colleague support and work–life balance (H4), underscoring the crucial role of job satisfaction in enhancing overall work–life integration. However, not all hypotheses were supported. The anticipated associations of leader support with work–life balance (H1b) and job satisfaction (H2b), as well as its mediation by job satisfaction between leader support and work–life balance (H5), were not significant. These findings open new perspectives on the relationship between support dynamics and remote work.

First, the results demonstrate a significant total mediating effect of job satisfaction in the relationship between support from colleagues and work–life balance. This finding underlines the importance of supportive interpersonal relationships within the workplace context. It suggests that when remote workers perceive a high level of support from their colleagues, they are more likely to experience greater job satisfaction, which in turn contributes to a better balance between work and personal life. This aligns with previous research highlighting the positive impact of social support on various work-related outcomes [55,56,57,58,59], as well as the importance of social support in remote work arrangements [5,8]. This effect may be explained in the light of the Conservation of Resources theory [60], according to which the resources gathered in a life domain (e.g., work) have a positive effect in other life domains (e.g., private life), even through the cross-over effect, namely, an emotional contagion effect occurring between individuals working together. Thus, it is likely that feeling supported by one’s coworkers (sharing the same work conditions, demands, and tasks and being able to receive support in dealing with it all) provides emotional and cognitive resources to employees so that they are more proactive and effective in dealing with private life demands and roles as well. Given the positive value of this connection, the broaden-and-build theory of positive emotions [40] can contribute to the interpretation of the findings as well. The positive, supportive interactions occurring with colleagues can foster better thoughts and behaviors so that employees not only feel more satisfied with their job experience but also have more cognitive and behavioral resources to put into place in their private lives. This study extends this research by showing that job satisfaction is a crucial mechanism through which social support impacts work–life balance among remote workers.

It is worth noting that this study found no significant direct link between support from leaders and work–life balance in remote workers. Contrary to initial hypotheses, findings did not confirm a direct link between support from leaders and work–life balance. Moreover, job satisfaction did not mediate this relationship as it did with support from colleagues. This result is somewhat surprising, as previous research has shown that leadership support can have a positive impact on employee well-being and work–life balance in traditional work arrangements (e.g., [61,62]).

One plausible interpretation is the nature of leadership within remote work environments. Unlike traditional office settings where leaders may exert more direct influence through face-to-face interactions, the dynamics of remote leadership often rely heavily on digital communication channels. This shift may impact the perceived effectiveness of leadership support in facilitating work–life balance among remote workers. Some studies showed, indeed, that structural forms of support may be particularly effective for remote employees, as they are in specific need of technical, information, and task-related support, not being able to benefit from the in-site interactions (e.g., [63]). Future research could explore the ways in which leaders can provide effective support to their remote employees and how these translate into perceived work–life balance outcomes. Another plausible explanation is that employees may require additional resources to feel engaged in their work, thereby enhancing the impact of leadership support practices on their work–life balance [64].

Furthermore, the absence of a direct relationship between leadership support and work–life balance underscores the multifaceted nature of this construct. While interpersonal support from colleagues may directly influence job satisfaction and, subsequently, work–life balance, the role of leadership support may be more indirect or contingent upon other factors such as organizational policies, communication practices, and role clarity. For instance, literature on remote working indicates that there are significant differences in the use of self-regulatory strategies to balance work and non-work responsibilities between younger and older workers [65].

This highlights the importance of considering age and job longevity as influential factors in understanding these distinct approaches.

Exploring these contextual factors in future studies could provide a more comprehensive understanding of the mechanisms underlying the relationship between leadership support and work–life balance among remote workers.

### 4.1. Practical Implications

These findings have several practical implications for organizations that employ remote workers. First, they highlight the importance of providing social support to remote workers to promote their well-being and work–life balance. Organizations can provide this support by fostering a culture of collaboration and communication among remote workers and by encouraging employees to develop strong social connections with their colleagues. This could involve facilitating team-building activities and providing platforms for informal communication. By investing in these initiatives, organizations can help mitigate the potential feelings of isolation and promote a healthier work–life balance for their remote workforce.

Additionally, this study underscores the importance of promoting job satisfaction among remote workers, as this is a crucial mechanism through which social support can impact work–life balance. Organizations should prioritize efforts to enhance job satisfaction through factors such as autonomy, recognition, and opportunities for skill development. By doing so, they can positively impact the well-being of remote workers and ultimately improve overall work–life balance within the remote work context.

Furthermore, the insignificance of leadership support within this model indicates that conventional leadership practices may not be as effective in the context of remote work. Therefore, organizations that adopt remote work should be mindful of the substantial variances between traditional and remote work environments, prompting the need for tailored management practices specifically designed for remote employees. This observation underscores the importance of developing new managerial strategies that account for the unique dynamics of remote work, ensuring that the potential of remote teams is fully realized. These findings have significant practical implications for organizations seeking to optimize their remote work arrangements and underscore the necessity for a shift in managerial approaches to accommodate the distinct requirements of remote work settings.

### 4.2. Practical Implications for Remote Workers

These findings have practical implications for different categories of stakeholders. Remote workers can utilize the findings of this study to advocate for the implementation of supportive policies and practices within their organizations. By leveraging the insights provided, remote employees can emphasize the importance of peer support and job satisfaction in contributing to a more fulfilling remote work experience. Understanding the significance of social connections and job satisfaction, employees can communicate the need for collaborative activities, informal communication platforms, and factors such as autonomy, recognition, and opportunities for skill development to enhance their well-being and work–life balance.

### 4.3. Practical Implications for Managers

Managers can use these findings to tailor their management practices specifically for remote employees. With the insignificance of traditional leadership support indicated in the study, managers need to recognize the substantial variances between traditional and remote work environments, prompting the need for new managerial strategies. By fostering a culture of collaboration and communication among remote workers and prioritizing efforts to enhance job satisfaction through autonomy, recognition, and skill development opportunities, managers can create a more supportive and conducive environment for remote work, ultimately realizing the potential of their remote teams.

### 4.4. Practical Implications for Organizations

Likewise, organizations can adopt the insights from this study to optimize their remote work arrangements. By investing in initiatives that promote social support, such as team-building activities and platforms for informal communication, organizations can help mitigate the potential feelings of isolation and promote a healthier work–life balance for their remote workforce. Furthermore, by understanding the necessity for tailored management practices specifically designed for remote employees, organizations can develop new managerial strategies that account for the unique dynamics of remote work, ensuring that the potential of remote teams is fully realized.

Overall, organizations can create a more supportive and conducive environment for remote work, leading to enhanced employee well-being and work–life balance by recognizing the importance of social support and job satisfaction.

## 5. Limitations

There are several limitations to consider when interpreting the findings of this study. First, the study relied on self-reported measures, which may be subject to biases and inaccuracies. For example, remote workers may have over-reported their levels of social support or job satisfaction due to social desirability bias or may have underreported their work–life balance issues due to perceived stigma.

Second, the study used a cross-sectional design, which limits the ability to draw causal conclusions. While a mediating effect of job satisfaction in the link between social support from colleagues and work–life balance emerged from the SEM model, it is possible that other variables not considered in this study may also impact this link. Future research could use longitudinal designs to explore these relationships further.

Third, the study sample consisted of remote workers from a specific industry (i.e., prevalently from service-based organizations) and geographic location (i.e., Italy), which may limit the generalizability of the findings to other populations. Future research could include more diverse samples to increase the generalizability of the findings.

Finally, the study did not consider other potential factors that may impact work–life balance in remote work arrangements, such as technology use, work demands, and home environment. Future research could explore these factors to gain a more comprehensive understanding of the factors that impact work–life balance in remote work arrangements.

## 6. Conclusions

In conclusion, the results of this study provide valuable insights into the factors that impact work–life balance among remote workers. Although it is essential to acknowledge the study’s limitations when interpreting the outcomes, the findings suggest that social support from colleagues plays a crucial role in nurturing work–life balance among remote workers. Furthermore, the study indicates that job satisfaction acts as a mediating factor in this relationship. As a result, these findings emphasize the significance of offering social support to remote workers and fostering job satisfaction as integral strategies for enhancing employee well-being and work–life balance within remote work environments.

It is evident that organizations should take into account these findings when formulating policies and support systems for remote workers. By recognizing the influence of social support and job satisfaction on work–life balance, organizations can tailor their approaches to better meet the needs of remote employees, ultimately contributing to improved well-being and productivity within the remote work setting. Furthermore, organizations should prioritize exploring innovative leadership-based support practices that diverge from traditional approaches and consider the peculiarities of remote work. These conclusions underscore the necessity for a holistic understanding of the factors impacting remote workers and the importance of integrating supportive measures into organizational frameworks to promote a healthier and more balanced work–life experience for remote employees.

The findings of this study have important implications for different stakeholders. Remote workers can use the insights to advocate for supportive policies and practices, emphasizing the importance of peer support and job satisfaction. Managers can tailor their practices for remote employees, fostering a culture of collaboration and communication. Organizations can optimize their remote work arrangements by investing in initiatives that promote social support and understanding the necessity for tailored management practices. Overall, these implications can lead to enhanced employee well-being and work–life balance.

## Figures and Tables

**Table 2:** Discriminant validity (Fornell–Larcker criterion).

	SUPP_COLL	LEAD_SUPPORT	JS	WLBALANCE_DEF
SUPP_COLL	0.650			
LEAD_SUPPORT	0.293	0.828		
JS	0.104	0.039	0.873	
WLBALANCE_DEF	0.044	0.025	0.432	-

**Table 3:** Mean, standard deviation, and correlations between variables.

	M	SD	SUPP_COLL	LEAD_SUPPORT	JS	WLBALANCE_DEF
SUPP_COLL	3.45	0.802	-			
LEAD_SUPPORT	3.70	0.999	0.541 ***	-		
JS	3.45	1.02	0.323 ***	0.197 ***	-	
WLBALANCE_DEF	3.73	1.26	0.209 ***	0.157 ***	0.657 ***	-

*** *p* ≤ 0.001.

## Data Availability

Data are available upon request.
